# Superexpressão Gênica PTEN em Tecidos Miocárdicos de Pacientes de Cirurgia de Revascularização Miocárdica

**DOI:** 10.36660/abc.20220169

**Published:** 2023-03-27

**Authors:** Reyhan Tahtasakal, Elif Funda Sener, Nesrin Delibasi, Zuhal Hamurcu, Ecmel Mehmetbeyoglu, Keziban Korkmaz Bayram, Isin Gunes, Dincer Goksuluk, Omer Naci Emirogullari

**Affiliations:** 1 Erciyes University Medical Faculty Department of Medical Biology Kayseri Turquia Erciyes University Medical Faculty Department of Medical Biology, Kayseri – Turquia; 2 Erciyes University Genome and Stem Cell Center Kayseri Turquia Erciyes University Genome and Stem Cell Center, Kayseri – Turquia; 3 Ankara Yildirim Beyazit University Medical Faculty Department of Medical Genetics Ankara Turquia Ankara Yildirim Beyazit University Medical Faculty Department of Medical Genetics, Ankara – Turquia; 4 Erciyes University Medical Faculty Department of Anesthesiology and Reanimation Kayseri Turquia Erciyes University Medical Faculty Department of Anesthesiology and Reanimation, Kayseri – Turquia; 5 Erciyes University Medical Faculty Department of Department of Biostatistics and Medical Informatics Kayseri Turquia Erciyes University Medical Faculty Department of Department of Biostatistics and Medical Informatics, Kayseri – Turquia; 6 Erciyes University Medical Faculty Department of Cardiovascular Surgery Kayseri Turquia Erciyes University Medical Faculty Department of Cardiovascular Surgery, Kayseri – Turquia

**Keywords:** Revascularização Miocárdica, Miocárdio, Expressão Gênica, PTEN

## Abstract

**Fundamento:**

A doença arterial coronariana é um distúrbio complexo que causa morte em todo o mundo. Um dos genes envolvidos no desenvolvimento dessa doença pode ser o PTEN.

**Objetivos:**

Nosso objetivo foi investigar a expressão gênica e proteica do PTEN em amostras de tecido e sangue retiradas de pacientes submetidos à cirurgia de revascularização miocárdica.

**Métodos:**

Foram realizados estudos moleculares no Centro de estudos do genoma humano e células-tronco da Universidade Erciyes (GENKOK). Amostras do apêndice atrial direito e de sangue foram coletadas da veia central de 22 pacientes submetidos à cirurgia de revascularização miocárdica antes de iniciar e terminar a circulação extracorpórea. A expressão do PTEN foi determinada usando PCR quantitativo em tempo real e análise de Western Blot. O nível de significância aceito foi de p<0,05.

**Resultados:**

Não houve diferença significativa na expressão gênica do PTEN em amostras de sangue coletadas antes e depois da circulação extracorpórea. Entretanto, foi observado um aumento substancial nos níveis de expressão gênica e proteica de P-PTEN e PTEN nas amostras de tecido. A expressão gênica miocárdica PTEN aumentou significativamente ao final da circulação extracorpórea. A expressão gênica do PTEN no período pós-circulação extracorpórea aumentou em comparação com o período pré-circulação extracorpórea, mas não foi um aumento significativo em comparação com sujeitos saudáveis do grupo de controle.

**Conclusão:**

Este estudo revelou pela primeira vez o papel do gene PTEN analisando a expressão de mRNA e de proteína em pacientes com revascularização miocárdica, que se manifesta tanto em tecido miocárdico quanto em amostras de sangue. O aumento dos níveis de PTEN pode ser um marcador no tecido miocárdico para pacientes com doença arterial coronariana.

**Figure:**
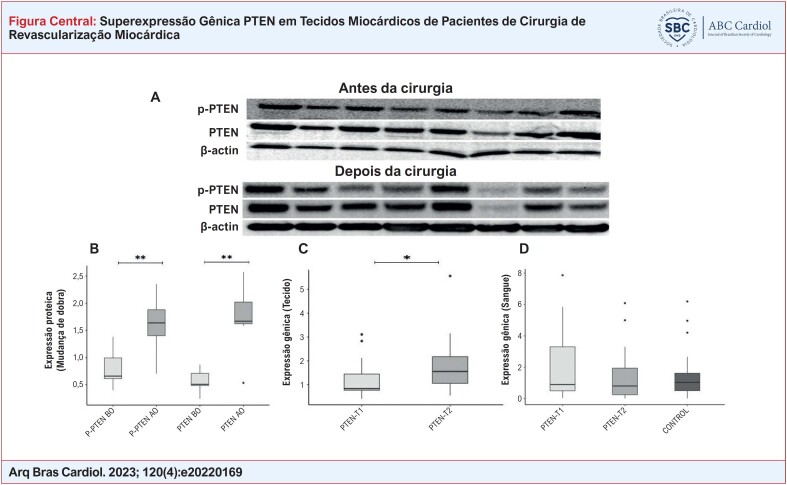
Resultados de expressão gênica e Western Blot de pacientes com DAC. Imagens de membrana dos níveis de proteína PTEN e P-PTEN determinados por anticorpos específicos em tecidos miocárdicos de pacientes do sexo feminino/masculino (A). Achados de expressão proteica de PTEN e P-PTEN em tecido miocárdico retirado de pacientes antes e após a cirurgia. A β-actina foi usada como controle de carregamento (B). A expressão gênica do PTEN foi determinada por PCR quantitativo em tempo real (qRT-PCR) em tecidos miocárdicos retirados de pacientes em 2 momentos diferentes (C). A expressão gênica do PTEN foi determinada por PCR quantitativo em tempo real (qRT-PCR) em amostras de sangue colhidas de pacientes em 2 momentos diferentes e em amostras de sangue de controle (D). Comparações: P-PTEN AC vs. P-PTEN DC, PTEN AC vs. PTEN DC, e PTEN-T1 vs. PTEN-T2 (em amostras de sangue e tecido separadamente) via teste Wilcoxon Signed Rand; PTEN-T1 vs. Controle e PTEN-T2 vs. Controle via teste U de Mann-Whitney (*p<0,05, **p<0,001). (T1: antes da circulação extracorpórea, T2: depois da circulação extracorpórea).

## Introdução

Uma das causas mais comuns de morte em homens e mulheres em todo o mundo são as doenças cardiovasculares,^1^ e uma das mais importantes é a doença arterial coronariana (DAC), que é afetada por muitos fatores.^2^ Fatores genéticos, bem como fatores ambientais, desempenham um papel essencial nas DAC.^3^ O mecanismo genético subjacente não é totalmente compreendido, mas estima-se que a herança possa ter uma efetividade de 40 a 60% na doença arterial coronariana. Estudos de associação genômica ampla (GWAS) revelaram que mais de 30 genes foram associados às DAC.^4 , 5^

A fosfatase homóloga à tensina (o PTEN) está localizada no cromossomo 10. Ele regula negativamente os níveis intracelulares de fosfatidilinositol-3,4,5-trifosfato nas células e atua como um supressor tumoral ao regular negativamente a via de sinalização AKT/PKB.^6 , 7^ O PTEN participa da via de progressão do ciclo celular, transdução de sinal, apoptose, reparo do DNA, crescimento e metabolismo. Ele também está associado à aterosclerose.^8^ O PTEN também pode desempenhar um papel no desenvolvimento e formação das DAC. A via PI3K/Akt protege o miocárdio em todas as espécies, especialmente contra danos de isquemia-reperfusão.^9^ A inativação do PTEN ativa a via PI3K/Akt, diminui a área de infarto, inibe a apoptose e aumenta a sobrevida. Ela também melhora o dano cardíaco pós-isquemia-reperfusão.^10^ Além disso, a inativação do PTEN cardioespecífico protege contra a fibrose miocárdica e a insuficiência cardíaca em um modelo de rato hipertenso.^11^ O PTEN é importante nas doenças cardíacas isquêmicas associadas a diabetes e obesidade.^12^ Portanto, garantir a inativação do PTEN também pode levar ao aumento da sobrevida miocárdica após um episódio isquêmico.^13^ A diminuição do nível de PTEN está associada à hipertrofia e à remodelação do tecido cardíaco.^14^ Além disso, demonstrou-se que o PTEN desempenha um papel na regulação do tamanho e da contração dos cardiomiócitos.^15^ Ressalta-se que o PTEN é extremamente importante na regulação do equilíbrio entre a morte e a sobrevida dos cardiomiócitos.^16^ Entretanto, a ausência prolongada de PTEN do miocárdio está associada à hipertrofia miocárdica.^15^

Portanto, este estudo enfocou as alterações na expressão de PTEN em amostras do tecido miocárdico e de sangue coletadas de pacientes submetidos à cirurgia de revascularização miocárdica durante e após a cirurgia. Para enfatizar a importância do gene PTEN no prognóstico das DAC, utilizamos amostras de sangue e de miocárdio em momentos diferentes da cirurgia.

## Métodos

### Seleção de pacientes e de grupo de controle

**Figura 1:**
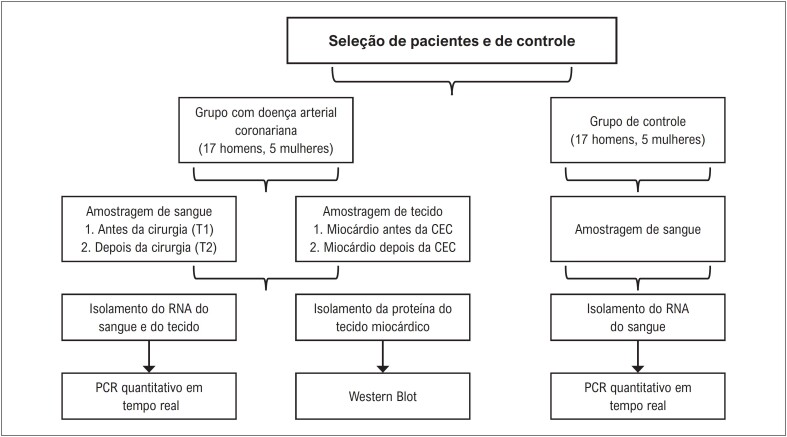
Grupo de controle, critérios do paciente e desenho do estudo. CEC: circulação extracorpórea. RNA: ácido ribonucleico; PCR: Reação em Cadeia da Polimerase

O conselho institucional de ética humana da Universidade Erciyes aprovou este estudo (2016/577; 08.11.2016). Foram incluídos no estudo vinte e dois pacientes com DAC tratados no Departamento de Cirurgia Cardiovascular da Universidade de Erciyes entre 2016 e 2017 ( Figura 1 ). O grupo de pacientes era composto de 5 mulheres e 17 homens (faixas etárias de 39 a 81 anos). Apenas pacientes com angina estável foram incluídos no estudo. Também inscrevemos 22 sujeitos saudáveis do grupo de controle para comparar os níveis sanguíneos da expressão gênica do PTEN. O grupo de controle foi formado por 5 mulheres e 17 homens saudáveis (faixas etárias de 26 a 69 anos). Idade e sexo foram pareados com o grupo de estudo. Os participantes deram consentimento informado por escrito para este estudo.

### Protocolo de anestesia

O mesmo protocolo de anestesia foi aplicado a todos os pacientes. Os pacientes que foram levados para a sala de cirurgia foram monitorados por ECG de 5 canais, oximetria de pulso, pressão arterial não invasiva, oximetria cerebral e entropia. Após injeção de 1,5 mg de midazolam e 50 microgramas de fentanila, um cateter arterial invasivo foi colocado na artéria radial e foi realizada uma medição invasiva da pressão arterial. Após a pré-oxigenação, foram administrados 1 mg/kg de propofol, 10 microgramas/kg de fentanila e 1 mg/kg de rocurônio na indução. Em seguida, pacientes com bons valores de entropia foram intubados. Foi inserido um cateter venoso central guiado por ultrassom. Foi realizada uma infusão de ácido tranexâmico na dose de 15 mg/kg em 1 hora, e mantida a partir de 1,5 mg/kg/h durante todo o caso. Foram usadas 10 microgramas/kg/h de fentanil, 4 mg/kg/h de propofol e 1 mg/kg/h de rocurônio no controle da anestesia. A profundidade da anestesia foi ajustada entre 45 e 60 entropias. Se necessário, o desflurano foi usado como agente inalatório. As configurações do ventilador foram TV 6 ml/kg, frequência respiratória, 12/min, PEEP de 5 cm/volume de água, com o modo de controle de volume aplicado. Caso houvesse uma redução da oximetria cerebral, as intervenções necessárias eram feitas.

### Período da cirurgia e da circulação extracorpórea

A mesma equipe de cirurgiões realizou todas as cirurgias. O procedimento cirúrgico incluiu esternotomia mediana e circulação extracorpórea (CEC) em todos os casos. CEC foi estabelecida pela canulação da aorta ascendente e uma única cânula de dois estágios no átrio direito. A proteção miocárdica foi feita com hipotermia moderada (28-32ºC), resfriamento tópico com solução salina e doses intermitentes (a cada 20 min) de cardioplegia sanguínea hipotérmica anterógrada. A cardioplegia sanguínea a quente foi administrada imediatamente antes da liberação do pinçamento aórtico. Todas as anastomoses coronárias distais foram construídas durante um único período de pinçamento aórtico, e as anastomoses proximais foram construídas sobre uma pinça de oclusão parcial durante o aquecimento. A substituição da valva aórtica foi realizada juntamente com a cirurgia de revascularização miocárdica em um caso.

#### Amostragem de tecidos e de sangue

Foram obtidas amostras de sangue da veia central dos pacientes em 2 momentos diferentes, antes e depois da cirurgia. Dois tecidos miocárdicos foram retirados 2-3 milímetros do átrio direito de cada paciente antes do início da CEC (T1) e ao final da CEC (T2). Foram obtidas amostras de sangue total do átrio direito de cada paciente antes do início da CEC (T1) e ao final da CEC (T2). O tecido ideal é o átrio direito como possível biomarcador no perfil proteômico do miocárdio em cirurgia de revascularização miocárdica, troca de válvula cardíaca e outros pacientes de cirurgia cardíaca.^17^ Portanto, o átrio direito foi a preferência em nosso estudo. Foram obtidas amostras de sangue dos sujeitos saudáveis do grupo de controle para comparar a expressão gênica do *PTEN* em pacientes. Todas as amostras foram imediatamente transferidas para o GENKOK para análise posterior.

#### Análise de PCR quantitativo e em tempo real do gene PTEN

Todos os estudos moleculares foram realizados no Centro de estudos do genoma humano e células-tronco da Universidade Erciyes (GENKOK). O RNA total foi isolado de todas as amostras de sangue e tecidos do miocárdio usando PureZol (Bio-Rad, Hercules, CA). As concentrações de RNA foram medidas usando um espectrofotômetro NanoDrop ND-1000 (NanoDrop Technologies, Inc., Rockland, DE, EUA). Foram usados dois indicadores, A260/A280 e A260/A230. A razão de absorbância foi de A260/A280, e A260/A230 foi um indicador de contaminação de proteína, contaminação de sal caotrópico, polissacarídeo e/ou fenol. A transcrição reversa do RNA (1 *μ* g) foi realizada usando o kit First Strand cDNA Synthesis (Roche Diagnostics GmbH, Mannheim, Alemanha) de acordo com as instruções do fabricante. A mistura de reação foi incubada a 65°C por 10 minutos, a 29°C por 10 minutos, a 48°C por 60 minutos, a 85°C por 5 minutos e depois a 4°C por 5 minutos. Aproximadamente 5 *μ* l de cada cDNA foram usados para análise de PCR. As reações do ensaio qPCR foram realizadas usando LightCycler 480 Probes Master e Primer/Probes (Roche Diagnostics GmbH) em um volume de reação de 20 *μ* l. As reações foram executadas em duplicata e o qRT-PCR foi realizado com o instrumento Light Cycler 480 II (Roche, Alemanha). As condições do ciclo foram as seguintes: desnaturação inicial a 95°C por 10 minutos, seguida de 10 segundos a 95°C, 30 segundos a 60°C e 60 segundos a 72°C, com as 3 etapas sendo de 45 ciclos. A última etapa ocorrerá em um ciclo a 40°C por 30 segundos para concluir a reação. Beta-actina (ACTB) foi selecionado como um gene de manutenção neste estudo. As mudanças na expressão gênica foram determinadas pelo método 2^-ΔΔ^Ct de quantificação relativa.

#### Análise Western Blot

As proteínas totais foram extraídas de dois tecidos do átrio direito dissecados usando protocolos padrão.^18^ A concentração total de proteína de cada amostra foi determinada com um kit de ensaio de proteína compatível com detergente (kit DC; Bio-Rad, Hercules, CA). Cada amostra contendo 40 µg de proteína total foi analisada por eletroforese em gel de gradiente de 4-20% de dodecil sulfato de sódio (SDS)-poliacrilamida. A separação das proteínas foi realizada e transferida para membranas de difluoreto de polivinilideno. As membranas foram tratadas com 0,1 Triton X-100, solução salina tamponada com Tris com Tween 20 (TBS-T) com 5% de leite em pó por um tempo específico. Foram examinados anticorpos PTEN primários e p-PTEN da marca Cell Signaling Technology. Após a lavagem com TBS-T, as membranas foram incubadas com um dos anticorpos secundários anticoelho ou anticamundongo (Biorad) apropriado para nosso estudo. β-actina e α/β Tubulina (primária e secundária) foram usadas como controle de carregamento. A detecção de quimioluminescência de membrana foi então realizada usando Clarity Western ECL Substrate (Biorad). As manchas foram visualizadas com um Chemidoc MP Imaging System (Biorad). Usando o programa aplicativo, o gerador de imagens foi medido com um densitômetro (Alpha Innotech, San Leandro, CA).

### Análise estatística

A análise dos dados foi realizada usando o GraphPad Prism (versão 8.01). A normalidade dos dados numéricos foi avaliada usando abordagens gráficas (gráfico Q-Q, histograma, etc.) e analíticas (teste de normalidade de Shapiro-Wilk). Resumimos dados numéricos (contínuos) (por exemplo, idade, peso, altura, níveis de expressão gênica de PTEN, tempos de pinçamento e revascularização, CK-MB e TnT) usando a média e o desvio padrão em dados normalmente distribuídos e mediana e interquartil em dados não normalmente distribuídos. As variáveis categóricas (por exemplo, sexo, tabagismo, hipertensão e diabetes, e o tipo de cirurgia) foram resumidas com frequências e porcentagens. Antes e depois da cirurgia, os níveis de expressão gênica medidos no tecido e no sangue foram comparados usando o teste de Wilcoxon, uma vez que esses valores não tinham distribuição normal. Da mesma forma, os níveis de expressão proteica antes e depois das amostras de tecido foram comparados usando o teste de Wilcoxon. Os níveis de expressão gênica em relação ao grupo de controle foram comparados em amostras de sangue usando o teste U de Mann-Whitney. Por último, calculamos os coeficientes de correlação de Spearman para explorar a relação entre os achados clínicos e laboratoriais do gene PTEN e a relação entre o tempo de pinçamento e o tempo total de revascularização. O nível de significância estatística foi definido como p<0,05 em todas as análises.

## Resultados

### Diagnóstico e características clínicas dos pacientes

Investigamos a expressão de mRNA e proteína do PTEN em grupos de controle e 22 pessoas com diagnóstico de DAC. A organização do estudo é apresentada na Figura 1 . No grupo de estudo, havia 17 homens (77,2%) e 5 (22,8%) mulheres. A faixa etária do grupo de estudo foi de 39 a 81 anos. Todos os achados clínicos e o tipo de cirurgia estão resumidos na Tabela 1 .

**Tabela 1:** Características clínicas e demográficas dos pacientes

Características clínicas		
Idade (39-81)		59,8±9,8
**Sexo**		
	Masculino (n,%)	17 (77,2%)
	Feminino (n,%)	5 (22,8%)
Diabetes (n)		6
Hipertensão, (n)		5
Tabagismo, (n)		9
Altura (cm)		165,6±8,6
Peso (kg)		79,2±13,5
ASC (m²)		1,8±0,2
FE (%)		49,5±7,2
Pinçamento (min)		59,8±14,3
Revascularização total (min)		133±42,5
Cirurgia		
	CABGx1 (n)	1
	CABGx2 (n)	2
	CABGx3 (n)	10
	CABGx4 (n)	6
	CABGx5 (n)	2
	SVA+CABG (n)	1

ASC: área da superfície corporal; CK-MB: creatina Quinase banda miocárdica; HDL: lipoproteína de alta densidade; LDL: lipoproteína de baixa densidade; FE: fração de ejeção; CABG: cirurgia de revascularização miocárdica; SVA: substituição da válvula aórtica.

### Achados laboratoriais gerais

**Figura 2:**
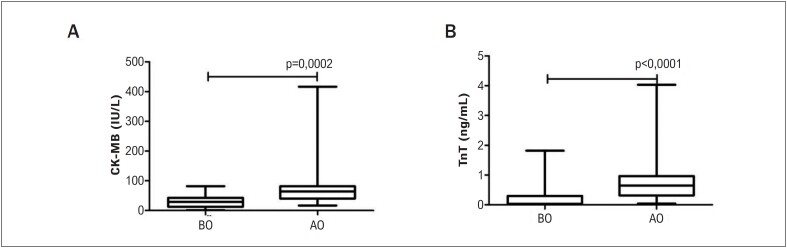
Achados bioquímicos dos pacientes. A) Níveis de CK-MB B) Níveis de troponina (Tnt). AC: antes da cirurgia, DC: depois da cirurgia. A análise estatística foi realizada por meio do teste de Wilcoxon para comparar os níveis dos achados bioquímicos antes e depois da cirurgia.

Os níveis da troponina T (TnT) e da enzima CK-MB foram medidos na amostra de sangue dos pacientes ( Figuras 2A e 2B ). Ambos os níveis de enzimas diferiram significativamente antes e depois da operação (p<0,0001 e p=0,0002, respectivamente).

### Níveis de expressão de PTEN

Primeiro, comparamos amostras de sangue pré (T1) e pós-operatórias (T2) da expressão gênica do PTEN, mas esse achado não foi estatisticamente significativo (p=0,1607). Em seguida, comparamos os valores pré-operatórios das expressões gênicas do PTEN com sujeitos saudáveis do grupo de controle, que também não foi significativo (p=0,9846) (Ilustração central). Por outro lado, a expressão de PTEN em amostras de miocárdio de pacientes aumentou significativamente após a CEC em comparação com antes da CEC (p=0,0417) (Ilustração central). Os resultados de nossos Western Blots mostraram que a expressão proteica de PTEN no tecido aumentou drasticamente após os dados de expressão gênica. A expressão proteica de p-PTEN também foi significativamente aumentada em amostras de tecido miocárdico após a CEC (Ilustração central). A mediana, o quartil e os valores de p nos dados de expressão gênica do PTEN em tecidos pré e pós-operatórios, sangue e amostras de sangue de controle são mostrados na Tabela 4 , e os valores medianos, interquartis e de p nos dados de expressão proteica são mostrados na Tabela 5 .

### Análise de correlação

De acordo com nossa análise de correlação, houve uma correlação moderada entre os valores pós-operatórios de CK-MB e TnT ( Tabela 2 ). As demais análises de correlação foram insignificantes, como idade, TnT e CK-MB com a expressão gênica do PTEN (r=0,1304; dados não apresentados).

**Tabela 2:** Análise de correlação de Spearman dos achados clínicos e laboratoriais do gene *PTEN*

	CK-MB T1	CK-MB T2	TnT T1	TnT T2	PTEN T1 mRNA	PTEN T2 mRNA
CK-MB T1	1,000					
CK-MB T2	0,411	1,000				
TnT T1	0,395	0,125	1,000			
TnT T2	0,422	**0,663** *	0,236	1,000		
PTEN T1 mRNA	-0,125	0,328	0,230	0,250	1,000	
PTEN T2 mRNA	-0,038	0,110	-0,023	0,322	**0,557** *	1,000

* p<0,05

Além disso, quando a análise de correlação foi feita entre a expressão de PTEN e P-PTEN e pinçamento e tempo total de revascularização, identificou-se que, embora haja uma relação estatisticamente insignificante negativa entre PTEN e P-PTEN e pinçamento, há uma relação estatisticamente insignificante positiva entre PTEN e P-PTEN e o tempo total de revascularização (dados não mostrados). Existe apenas uma relação positiva entre PTEN e P-PTEN, e essa relação é estatisticamente significativa (r=0,90, p=0,02). ( Tabela 3 ).

**Tabela 3:** Correlação de Spearman para expressão proteica com tempo de pinçamento e tempo total de revascularização (* r= 0,90, p=0,02)

	P-PTEN	PTEN	Tempo de pinçamento (min)	Tempo total de revascularização (min)
P-PTEN	1,000			
PTEN	0,90*	1,000		
Tempo de pinçamento (min)	-0,53	-0,31	1,000	
Tempo total de revascularização (min)	0,17	0,28	0,21	1,000

**Tabela 4:** Demonstração de valores medianos, interquartis e p de variáveis contínuas não normalmente distribuídas nos dados de expressão gênica em amostras de tecido e sangue pré e pós-operatório e amostras de sangue de controle

	Expressão gênica do PTEN			Significância (Amostras de sangue) ‡	
	Sangue (n=22)	Tecido (n=22)	Controle (n=22)	Pré-Op vs. Controle	Pós-Op vs. Controle
**Pré-Op**	0,89 (0,47, 3,49)	0,83 (0,76, 1,50)	1,03 (0,41, 1,91)	0,814	0,742
**Pós-Op**	0,80 (0,20, 2,13)	1,55 (0,97, 2,23)			
Sig. (p) †	0,072	**0,053**	N.A.		

* Resumido usando mediana (quartis). N.A.: Não se aplica.

† Comparações pré-op versus pós-op pelo teste de Wilcoxon.

‡ Versus comparações de controle quanto a amostras de sangue via teste U de Mann-Whitney.

**Tabela 5:** Demonstração de valores medianos, interquartis e p de variáveis contínuas não normalmente distribuídas nos dados de expressão proteica em amostras de tecido e sangue pré e pós-operatório

Expressão proteica do PTEN		
**n=8**	**P-PTEN**	**PTEN**
**Pré-Op**	0,66 (0,61, 1,14)	0,51 (0,47, 0,84)
**Pós-Op**	1,64 (1,23, 2,10)	1,67 (1,58, 2,28)
**Sig. (p)**	**0,018**	**0,018**

* Resumido usando mediana (quartis) e comparado pelo teste de Wilcoxon.

## Discussão

Doenças cardiovasculares (DCV), como doença arterial coronariana (DAC), infarto do miocárdio (IM) e acidente vascular cerebral, são causas significativas de mortalidade e morbidade na população em geral em todo o mundo.^2^ A DAC é a causa mais comum de morte no mundo entre as doenças cardiovasculares. O PTEN tem um papel crítico na hipertrofia e sobrevida dos cardiomiócitos. Portanto, neste estudo, avaliamos a expressão gênica e proteica do PTEN em pacientes com DAC.

Mocanu et al. afirmaram que a via de sinalização PI3K/AKT é vital na proteção do miocárdio contra lesão de isquemia-reperfusão.^16^ Eles enfatizaram que o principal fator que leva a essa ativação é o gene PTEN. Além disso, o PTEN pode ser essencial em condições patológicas associadas a doenças cardíacas isquêmicas, como diabetes e obesidade.^12^ Portanto, a inativação do PTEN pode desempenhar um papel importante em condições patológicas associadas à doença cardíaca isquêmica.^13^

Li et al. investigaram a expressão de PTEN para explorar a relevância entre a expressão de PTEN e o desenvolvimento de DCC no tecido miocárdico de 16 pacientes mortos por imuno-histoquímica e qRT-PCR. A expressão proteica do PTEN no miocárdio foi significativamente menor em pacientes com doença cardíaca coronária em comparação com o grupo de controle saudável. Ao mesmo tempo, não houve diferença estatística na expressão de PTEN mRNA entre o grupo experimental e o de controle. Com esses resultados, eles concluíram que o PTEN pode estar envolvido na ocorrência e no desenvolvimento de DCC.^19^ Nosso estudo também mostrou que a expressão gênica e proteica do PTEN no tecido miocárdico aumentou após a revascularização. Pensamos que os resultados diferiam porque amostras de tecido pós-morte foram usadas no estudo mencionado acima.

As vias de sinalização PTEN/PI3K desempenham um papel crucial na patogênese da hipertrofia miocárdica. Em um estudo, descobriu-se que ela estava envolvida na remodelação miocárdica em 39 pacientes com insuficiência cardíaca congestiva (ICC). As expressões proteica do PTEN nos grupos com insuficiência cardíaca congestiva foram menores do que no grupo de controle e foram negativamente correlacionadas aos níveis de função cardíaca. Assim, eles revelaram que o PTEN pode desempenhar um papel regulador negativo no processo de remodelação miocárdica.^20^ A hipertrofia cardíaca, uma resposta adaptativa geral do coração, é um processo complexo no qual muitos genes atuam de maneira coordenada. Ela pode ser estimulada de várias maneiras, incluindo a via IGF-1/PI3K/Akt. Na via de sinalização PI3K, o PTEN pode ser um determinante crítico do crescimento de cardiomiócitos. Sob certas condições, pode causar apoptose, prevenir a hipertrofia e bloquear a sinalização do fator de crescimento.^14^ Em nosso estudo, a expressão gênica e proteica do PTEN em tecidos miocárdicos pré-operatórios aumentou significativamente após a cirurgia. Esse resultado nos mostra repetidamente que o PTEN desempenha um papel importante na reparação e aumento da sobrevivência celular, contratilidade miocárdica e viabilidade de cardiomiócitos em danos teciduais. Considerando todos esses efeitos, o PTEN será um importante marcador de prognóstico em pacientes com DAC. Um dos estudos recentes revelou que o PTEN desempenha um papel na progressão do infarto do miocárdio. Observou-se que os níveis séricos de PTEN estão aumentados em pacientes com infarto agudo do miocárdio, sugerindo que o PTEN pode ser usado como marcador preditivo de IM.^21 , 22^ Um dos estudos recentes é o de Feng et al. Em 2020, eles descobriram que o BPV como um inibidor de PTEN administrado aos camundongos melhorou a função dos vasos cardíacos após o infarto do miocárdio e consideraram que o inibidor de PTEN BPV poderia ser uma droga terapêutica candidata.^23^

A atividade do PTEN pode ser reduzida de duas maneiras: uma é conseguida pela inativação enzimática por fosforilação ou oxidação, e outra pela alteração do equilíbrio entre síntese e degradação do PTEN. A mudança na atividade PTEN é importante para manter o equilíbrio em muitos tipos de células. Também desempenha um papel vital na regulação do equilíbrio entre sobrevivência e morte em cardiomiócitos.^16^ A função do PTEN é regulada por diferentes modificações pós-traducionais, como fosforilação, acetilação, ubiquitinação e oxidação. O PTEN participa da modulação e estabilidade de suas funções supressoras de tumor. Também possui seis sítios de fosforilação envolvidos na compartimentalização subcelular.^8 , 24^ A região C-terminal do PTEN é composta por 218 aminoácidos e desempenha um papel na regulação da estabilidade e meia-vida da molécula. Essa região é rica em locais de fosforilação, e foi relatado que a fosforilação da região C-terminal do PTEN afeta a estabilidade e a função da proteína PTEN. Notavelmente, os sítios de fosforilação da caseína quinase 2 (CK2) no PTEN são conservados em espécies.^25^ Nosso estudo também corrobora essa ideia. Mostramos que o PTEN aumentou a fosforilação a partir do ponto S380 na cauda C. A fosforilação do resíduo S380 inibe a atividade catalítica do PTEN e estabiliza a proteína bloqueando a associação produtiva do domínio catalítico do PTEN com PI(3,4,5)P3 localizado na membrana.^25^

Demonstramos uma diferença significativa na expressão de PTEN dos tecidos miocárdicos durante a cirurgia. Em nosso estudo, a obtenção de amostras de sangue e tecidos em vários momentos da cirurgia foi difícil, mas diferenciou nosso trabalho dos demais. Sem o uso de pacientes saudáveis no grupo de controle, primeiro comparamos as amostras de tecido e sangue dentro do grupo, eliminando as diferenças individuais e possibilitando mostrar diferenças nos níveis de expressão na mesma ocasião. Em seguida, incluímos o grupo de controle para comparar os níveis de expressão de PTEN no sangue. A expressão gênica de PTEN no sangue pode ser reduzida por epigenética e outros mecanismos. Os efeitos da epigenética podem alterar a atividade de PTEN. Alguns miRNAs podem se ligar ao 3’UTR do PTEN (como o miR-21), alterar a proliferação de células endoteliais vasculares via PI3K-Akt e afetar as DAC.^26^ Alguns estudos anteriores^27^ sugeriram que o PTEN é regulado positivamente em células mononucleares do sangue periférico de pacientes com DAC. Isso é consistente com os achados do estudo de Zhang et al. sobre leucócitos do sangue periférico,^28^ e esses resultados do estudo corroboram nosso estudo. Nossos resultados diferiram dos níveis sanguíneos do estudo mencionado em comparação com os sujeitos saudáveis do grupo de controle. Os mesmos níveis de PTEN no sangue foram detectados no grupo de controle e nos pacientes.

O papel de TnI e TnT como marcadores proteicos de infarto do miocárdio foi proposto pela primeira vez há mais de 20 anos. A medição imunoquímica das concentrações de TnI ou TnT no sangue do paciente afastou os métodos tradicionais de diagnóstico de infarto do miocárdio.^29^ Os níveis de troponina e CK-MB referem-se a necrose e isquemia nos cardiomiócitos. A troponina T cardioespecífica (cTnT) é elevada no sangue após a injúria cardíaca.^30^ O aumento desses dados clínicos também está relacionado ao grau de isquemia. De acordo com a literatura, nosso estudo constatou que tanto os níveis de CK-MB quanto de troponina aumentaram significativamente após a cirurgia. Também determinamos uma correlação entre os níveis de CK-MB e TnT. Com base nesses achados, seria útil determinar a causa da diferença de troponina e CK-MB planejando estudos mais extensos e diferentes para identificar o dano estrutural no tecido miocárdico. Além das funções conhecidas da troponina, foi relatado que ela desempenha um papel na proliferação celular.^31^ Como mencionado acima, os níveis elevados de biomarcadores clínicos do miocárdio, tanto a expressão gênica quanto a proteica do PTEN, foram maiores após a cirurgia. Um aumento do PTEN como supressor de tumor devido ao aumento do nível de TnT também pode ser responsável por suprimir a proliferação de células danificadas.

Como em muitos sistemas, os miRNAs desempenham um papel essencial no sistema cardiovascular. Além de controlar as funções de células como cardiomiócitos, células musculares lisas, fibroblastos e células endoteliais, eles também são cruciais na fisiopatologia de doenças como infarto do miocárdio, hipertrofia, fibrose, insuficiência cardíaca, arritmia, inflamação e aterosclerose. Portanto, a investigação dos miRNAs associados ao PTEN também se beneficiará da expansão de nosso estudo e da aquisição de novos dados. Nesse sentido, nosso estudo é o primeiro na literatura. Entretanto, ao considerar as incógnitas sobre a via do PTEN, seria apropriado planejar novas investigações nessa área.

O PTEN funciona como um regulador crítico da hipertrofia e sobrevivência dos cardiomiócitos. A partir desse ponto de vista, investigamos a expressão gênica e proteica do PTEN em amostras de sangue e tecidos. Estudos utilizando sangue e tecidos miocárdicos são raros na literatura, e é um desafio realizá-los. Quando comparamos a expressão gênica do PTEN no sangue, não houve diferença entre a expressão do PTEN antes e depois da cirurgia. Também não foi encontrada nenhuma diferença quando os pacientes foram comparados com os do grupo de controle. Observamos aumento da expressão gênica e proteica no tecido após a cirurgia em comparação com antes da cirurgia. O aumento da expressão de PTEN no tecido miocárdico após a cirurgia em comparação com a amostra pré-operatória mostra repetidamente que o PTEN desempenha um papel vital na reparação do dano tecidual, na cicatrização e no aumento da sobrevida celular, da contratilidade miocárdica e da viabilidade dos cardiomiócitos. Considerando todos esses efeitos, o gene PTEN nos permitirá tomar uma medida prognóstica crítica na doença arterial coronariana nos próximos anos. Estudos moleculares nesta área são escassos, e acreditamos que nossas descobertas sobre o PTEN podem levar a estudos futuros. A limitação deste estudo é o número pequeno de pacientes. Além disso, nossos resultados podem ser corroborados pela investigação da relação entre parâmetros clínicos mais amplos e níveis de PTEN.

## Conclusão

O PTEN funciona como um regulador crítico da hipertrofia e sobrevivência dos cardiomiócitos. De acordo com os resultados obtidos neste estudo, pode-se sugerir que o gene PTEN pode ser um marcador nesse grupo de doenças, ampliando o número de pacientes para futuras investigações. Entretanto, ao considerar as incógnitas sobre a via do PTEN, seria apropriado planejar novas investigações nessa área. Estudos inovadores com amostras maiores podem ser planejados para concluir o real efeito da atividade do PTEN no miocárdio e aceitá-lo como marcador de prognóstico.
