# Supplementary material for: Superexpressão Gênica PTEN em Tecidos Miocárdicos de Pacientes de Cirurgia de Revascularização Miocárdica
Source: Arq Bras Cardiol. 2023 Mar 27;120(4):e20220169. [Article in Portuguese] doi: 10.36660/abc.20220169 (PMC10263415; doi:10.36660/abc.20220169)
Supplement: Translation

# Translation: Overexpression of the PTEN Gene in Myocardial Tissues of Coronary Bypass Surgery Patients

**Authors:** Reyhan Tahtasakal, Elif Funda Sener, Nesrin Delibasi, Zuhal Hamurcu, Ecmel Mehmetbeyoglu, Keziban Korkmaz Bayram, Isin Gunes, Dincer Goksuluk, Omer Naci Emirogullari

**Affiliations:** 1 Kayseri Turkey Erciyes University Medical Faculty Department of Medical Biology, Kayseri – Turkey; 2 Kayseri Turkey Erciyes University Genome and Stem Cell Center, Kayseri – Turkey; 3 Ankara Turkey Ankara Yildirim Beyazit University Medical Faculty Department of Medical Genetics, Ankara – Turkey; 4 Kayseri Turkey Erciyes University Medical Faculty Department of Anesthesiology and Reanimation, Kayseri – Turkey; 5 Kayseri Turkey Erciyes University Medical Faculty Department of Department of Biostatistics and Medical Informatics, Kayseri – Turkey; 6 Kayseri Turkey Erciyes University Medical Faculty Department of Cardiovascular Surgery, Kayseri – Turkey

**Keywords:** Coronary Bypass Surgery, Myocardium, Gene Expression, PTEN

## Abstract

**Background:**

Coronary artery disease is a complex disorder that causes death worldwide. One of the genes involved in developing this disease may be PTEN.

**Objectives:**

This study aimed to investigate the PTEN gene and protein expression in tissue and blood samples taken from coronary bypass surgery patients.

**Methods:**

Molecular studies were performed at Erciyes University Genome and Stem Cell Center (GENKOK). Right atrial appendage and blood samples were taken from the central vein of 22 coronary bypass surgery patients before starting and ending cardiopulmonary bypass. PTEN expression was determined using quantitative real-time PCR and western blot analysis. The significance level was accepted as p<0.05.

**Results:**

There was no significant difference in the PTEN gene expression in blood samples taken before and after cardiopulmonary bypass. However, a substantial increase in both protein and gene expression levels of P-PTEN and PTEN was observed in the tissue samples. Myocardial expression of the PTEN gene was significantly increased at the end of the cardiopulmonary bypass. PTEN gene expression in the post-cardiopulmonary bypass period was increased when compared to the pre-bypass period, but it was insignificant when compared to healthy controls.

**Conclusion:**

This study first revealed the role of the PTEN gene by analyzing both mRNA and protein expression in coronary bypass patients, appearing in both myocardial tissue and blood samples. Increased levels of PTEN may be a marker in myocardial tissue for patients with coronary artery disease.

**Figure:** Gene expression and western blot results of the patients with CAD. Membrane images of PTEN and P-PTEN protein levels determined using specific antibodies in myocardial tissues of female/male patients (A). PTEN and P-PTEN protein expression findings in myocardial tissue taken from patients before and after surgery. β-actin was used as a loading control (B). The expression of the PTEN gene was determined by quantitative real-time PCR (qRT-PCR) in myocardial tissues taken from patients at 2 different times (C). Expression of the PTEN gene was determined by quantitative real-time PCR (qRT-PCR) in blood samples taken from patients at 2 different times and in control blood samples (D). Comparisons: P-PTEN BO vs. P-PTEN AO, PTEN BO vs. PTEN AO, and PTEN-T1 vs. PTEN-T2 (in blood and tissue samples separately) via Wilcoxon Signed Rand test; PTEN-T1 vs. Control and PTEN-T2 vs. Control via Mann-Whitney U test (*p<0.05, **p<0.001). (T1: before cardiopulmonary bypass, T2: after cardiopulmonary bypass).

## Introduction

One of the most common causes of death in men and women worldwide is cardiovascular disease,^1^and one of the most important of these is coronary artery disease (CAD), which is affected by many factors.^2^Genetic factors, as well as environmental factors, play an essential role in CAD.^3^The underlying genetic mechanism is not fully understood, but it is estimated that 40-60% inheritance may be effective in coronary artery disease. Genome-wide association studies (GWAS) revealed that more than 30 genes were associated with CAD.^4 , 5^

Phosphatase and tensin homolog ( *PTEN* ) are located on chromosome 10. They negatively regulate intracellular phosphatidylinositol-3,4,5-trisphosphate levels in cells and act as a tumor suppressors by negatively regulating the AKT/PKB signaling pathway.^6 , 7^ *PTEN* participates in the pathway of cell cycle progression, signal transduction, apoptosis, DNA repair, growth, and metabolism. It is also associated with atherosclerosis.^8^ *PTEN* may also play a role in the development and formation of CAD. The PI3K/Akt pathway protects the myocardium in all species, especially against ischemia-reperfusion damage.^9^The inactivation of *PTEN* activates the PI3K/Akt pathway, decreases the infarct area, inhibits apoptosis, and enhances survival. It also improves post-ischemia-reperfusion heart damage.^10^Furthermore, the inactivation of cardiac-specific *PTEN* protects against myocardial fibrosis and cardiac failure in a hypertensive mouse model.^11^ *PTEN* is important in ischemic heart diseases associated with diabetes and obesity.^12^Therefore, providing *PTEN’s* inactivation may also increase myocardial survival following an ischemic episode.^13^The decrease in the *PTEN* level is associated with hypertrophy and the remodeling of cardiac tissues.^14^In addition, *PTEN* has also proven to play a role in regulating size and contraction in cardiomyocytes.^15^It is emphasized that *PTEN* is extremely important in regulating the balance between cardiomyocyte death and survival.^16^However, the long-term absence of *PTEN* from the myocardium is associated with myocardial hypertrophy.^15^

Therefore, this study focused on the changes in *PTEN* expression in myocardial tissue and blood samples collected from coronary artery bypass surgery patients during and after the surgery. To emphasize the importance of the *PTEN* gene in the prognosis of CAD, both myocardial and blood samples were used at different stages of surgery.

## Methods

### Patients and control selection

The institutional human ethics review board of Erciyes University approved this study (2016/577; 08.11.2016). Twenty-two patients with CAD treated at the Department of Cardiovascular Surgery of Erciyes University between 2016 and 2017 were included in the study ( Figure 1 ). The patient group consisted of 5 women and 17 men (age ranges 39-81 years). Only patients with stable angina were included in the study. Our study also enrolled 22 healthy controls to compare the blood levels of *PTEN* gene expression. Controls consisted of 5 healthy women and 17 healthy men (age ranges of 26-69 years). Age and gender were matched with the study group. The participants gave written informed consent for this study.

**Figure 1:** Control group, patient’s criteria, and study design. CPB: cardiopulmonary bypass. RNA: Ribonucleic Acid; PCR: Polymerase Chain Reaction.

### Anesthesia protocol

The same anesthesia protocol was applied to all patients. A 5-channel ECG, pulse oximetry, noninvasive blood pressure, cerebral oximetry, and entropy monitoring were performed on the patients who were taken to the operating room. After a 1.5 mg dormicum and 50 microgram fentanyl push were made, an invasive arterial catheter was placed in the radial artery, and an invasive blood pressure measurement was performed. After preoxygenation, 1 mg/kg propofol, 10 micrograms/kg fentanyl, 1 mg/kg rocuronium were made in the induction. Patients with good entropy values were then intubated. An ultrasound-guided central venous catheter was inserted. Tranexamic acid was infused at a dose of 15 mg/kg in 1 hour and was maintained at 1.5 mg/kg/h throughout the case; 10 micrograms/kg/h fentanyl, 4 mg/kg/h propofol and 1 mg/ kg/h rocuronium were used in anesthesia management. The depth of anesthesia was adjusted between 45 and 60 entropies. If necessary, desflurane was used as an inhalation agent. Ventilator settings TV 6 ml/kg, respiratory rate 12/min, PEEP of 5 cm/water volume applied volume control mode. If there was a decrease in cerebral oximetry, necessary interventions were performed.

### Operation and cardiopulmonary bypass period

The same team of surgeons performed all operations. The operative procedure included median sternotomy and cardiopulmonary bypass (CPB) in all cases. CPB was established by the cannulation of the ascending aorta and a single two-stage cannula in the right atrium. Myocardial protection was provided with moderate hypothermia (28-32ºC), topical cooling with saline solution, and intermittent doses (every 20 min) of antegrade hypothermic blood cardioplegia. Warm blood cardioplegia was given just before releasing the aortic cross-clamping. All distal coronary anastomoses were constructed during a single period of aortic cross-clamping, and proximal anastomoses were constructed over a partial occlusion clamp during warming. Aortic valve replacement was done together with coronary bypass surgery in one case.

#### Tissue and blood sampling

Blood samples were obtained from the central vein of the patients at 2 different times, before and after surgery. Two myocardial tissues were taken 2-3 millimeters from each patient’s right atrium before starting CPB (T1) and at the end of CPB (T2). Whole blood samples were obtained from each patient’s right atrium before starting CPB (T1) and at the end of CPB (T2). The ideal tissue is the right atrium as a potential biomarker in a myocardial proteomic profile in coronary bypass surgery, heart valve replacement, and other cardiac surgery patients.^17^Therefore, the right atrium was preferred in our study. Blood samples were taken from the healthy controls to compare *PTEN* gene expression in patients. All the samples were immediately transferred to GENKOK for further analysis.

#### Real-time quantitative PCR analysis for PTEN gene

All molecular studies were conducted at Erciyes University Genome and Stem Cell Center (GENKOK). Total RNA was isolated from all blood samples and myocardial tissues using PureZol (Bio-Rad, Hercules, CA). RNA concentrations were measured using a nanodrop ND-1000 spectrophotometer (NanoDrop Technologies, Inc., Rockland, DE, USA). Two indicators, A260/A280 and A260/A230, were used. The absorbance ratio was A260/A280, and A260/A230 was an indicator of protein contamination, chaotropic salt contamination, polysaccharide, and/or phenol. RNA (1 *μ* g) was reverse transcribed using the First Strand cDNA Synthesis kit (Roche Diagnostics GmbH, Mannheim, Germany) according to the manufacturer’s instructions. The reaction mixture was incubated at 65°C for 10 minutes, at 29°C for 10 minutes, at 48°C for 60 minutes, at 85°C for 5 minutes, and then at 4°C for 5 minutes. Approximately 5 *μ* l of each cDNA was used for PCR analysis. The qPCR assay reactions were performed using LightCycler 480 Probes Master and Primer/Probes (Roche Diagnostics GmbH) in a 20 *μ* l reaction volume. Reactions were run in duplicate, and qRT-PCR was performed with Light Cycler 480 II instrument (Roche, Germany). The cycle conditions were as follows: initial denaturation at 95°C for 10 minutes, followed by 10 seconds at 95°C, 30 seconds at 60°C, and 60 seconds at 72°C, with the 3 steps including 45 cycles. The last step will occur in a cycle at 40°C for 30 seconds to complete the reaction. Beta-actin ( *ACTB* ) was selected as a housekeeping gene in this study. The changes in the gene expression were determined by the 2^-ΔΔ^Ct method of relative quantification.

#### Western blot analysis

Total proteins were extracted from dissected two right atrium tissues using standard protocols.^18^Each sample’s total protein concentration was determined with a detergent-compatible protein assay kit (DC kit; Bio-Rad, Hercules, CA). Each sample containing 40 µg of total protein was analyzed by loading 4-20% gradient sodium dodecyl sulfate (SDS)-polyacrylamide gel electrophoresis. Protein separation was performed and transferred to polyvinylidene difluoride membranes. Membranes were treated with 0.1 Triton X-100, Tris-buffered saline with 5% dry milk-Tween 20 (TBS-T) for a specific time. Cell Signaling Technology brand primary PTEN antibody and p-PTEN were examined. After washing with TBS-T, membranes were incubated with one of the anti-rabbit or anti-mouse secondary antibody (Biorad) appropriate for our study. β-actin and α/β Tubulin (primary and secondary) were used as a loading control. Membrane chemiluminescence detection was then performed using Clarity Western ECL Substrate (Biorad). Blots were imaged with a Chemidoc MP Imaging System (Biorad). Using the application program, the imager was measured with a densitometer (Alpha Innotech, San Leandro, CA).

### Statistical analysis

Data analysis was performed using the GraphPad Prism (version 8.01). The normality of numerical data was assessed using graph (Q-Q plot, histogram, etc.) and analytical approaches (Shapiro-Wilk’s normality test). We summarized numerical (continuous) data (e.g., age, weight, height, gene expression levels of PTEN, clamp and bypass times, CK-MB, and TnT) using the mean and standard deviation in normally distributed data and median and interquartile in non-normally distributed data. Categorical variables (e.g., gender, smoking status, hypertension and diabetes status, and the operation type) were summarized with frequencies and percentages. Before and after the operation, gene expression levels measured from tissue and blood were compared when using the Wilcoxon test, since these values were not normally distributed. Likewise, before and after, protein expression levels of tissue samples were compared using the Wilcoxon test. The gene expression levels against the control group were compared in blood samples using the Mann-Whitney U test. Finally, we calculated Spearman correlation coefficients to explore the relationship between clinical and laboratory findings of the PTEN gene and the relationship between cross-clamp time and total bypass time. The statistical significance level was set at p<0.05 in all analyses.

## Results

### Patient’s diagnosis and clinical features

This study investigated mRNA and protein expression of PTEN in control groups and 22 people with a diagnosis of CAD. The organization of the study is shown in Figure 1 . In the study group, there were 17 males (77.2%) and 5 (22.8%) females. The age range of the study group was 39-81 years. All the clinical findings and the type of operation are summarized in Table 1 .

**Table 1:** Clinical and demographical characteristics of the patients

Clinical Features		
Age (39-81)		59.8±9.8
**Gender**		
	Male (n,%)	17 (77.2%)
	Female (n,%)	5 (22.8%)
Diabetes (n)		6
Hypertension (n)		5
Smoking (n)		9
Height (cm)		165.6±8.6
Weight (kg)		79.2±13.5
BSA (m²)		1.8±0.2
EF (%)		49.5±7.2	
Cross Clamp (min)		59.8±14.3	
Total Bypass (min)		133±42.5	
**Operation**		
	CABGx1 (n)	1
	CABGx2 (n)	2
	CABGx3 (n)	10
	CABGx4 (n)	6
	CABGx5 (n)	2
	AVR+CABG (n)	1

BSA: body surface area; CK-MB: creatine kinase myocardial band; HDL: high-density lipoprotein; LDL: low-density lipoprotein; EF: ejection fraction; CABG: coronary artery bypass graft surgery; AVR: aortic valve replacement.

### General laboratory findings

Troponin T (TnT) and CK-MB enzyme levels were measured in the blood sample of patients ( Figures 2A and 2B ). Both enzyme levels significantly differed before and after the operation (p<0.0001, p=0.0002, respectively).

**Figure 2:** Biochemical findings of the patients. A) CK-MB levels B) Troponin (Tnt) levels. BO: before an operation, AO: after an operation. Statistical analysis was performed using the Wilcoxon test to compare the levels of biochemical findings before and after surgery.

### Expression levels of PTEN

Our study first compared pre- (T1) and post-operative (T2) blood samples of *PTEN* gene expression, but this finding proved not to be statistically significant (p=0.1607). We then compared the pre-operative values of *PTEN* gene expressions with healthy controls, which also proved not to be significant (p=0.9846) (Central Illustration). On the other hand, the expression of *PTEN* in myocardium samples of patients significantly increased after CPB compared to before CPB (p=0.0417) (Central Illustration). Our western blot results showed that the protein expression of PTEN in the tissue increased dramatically following gene expression data. p-PTEN protein expression was also significantly increased in myocardial tissue samples after CPB (Central Illustration). Median, quartile, and p-values in *PTEN* gene expression data in pre- and postoperative tissue, as well as in blood and control blood samples, are shown in Table 4 , and median, interquartile, and p-values in protein expression data are shown in Table 5 .

### Correlation analysis

According to our correlation analysis, there was a moderate correlation between post-operative values of CK-MB and TnT ( Table 2 ). The other correlation analysis was insignificant, including age, TnT, and CK-MB with *PTEN* gene expression (r=0.1304; data not shown).

**Table 2:** Spearman Correlation analysis of the clinical and laboratory findings of *PTEN* gene

	T1 CK-MB	T2 CK-MB	T1 TnT	T2 TnT	T1 *PTEN* mRNA	T2 *PTEN* mRNA
T1 CK-MB	1.000					
T2 CK-MB	0.411	1.000				
T1 TnT	0.395	0.125	1.000			
T2 TnT	0.422	**0.663** *	0.236	1.000		
T1 PTEN mRNA	-0.125	0.328	0.230	0.250	1.000	
T2 PTEN mRNA	-0.038	0.110	-0.023	0.322	**0.557** *	1.000

* p<0.05

In addition, when correlation analysis was made between PTEN and P-PTEN expression and cross-clamp and total bypass time, while there is a negative statistically insignificant relationship between PTEN and P-PTEN and thr cross clamp, there is a positive statistically insignificant relationship between PTEN and P-PTEN and total bypass time (Data not shown). There is only a positive relationship between PTEN and P-PTEN, and this relationship is statistically significant (r=0.90, p=0.02) ( Table 3 ).

**Table 3:** Spearman Correlation for protein expression with cross clamp time and total bypass time (* r= 0.90, p=0.02)

	P-PTEN	PTEN	Cross Clamp Time (min)	Total Bypass Time (min)
P-PTEN	1.000			
PTEN	0.90*	1.000		
Cross Clamp Time (min)	-0.53	-0.31	1.000	
Total Bypass Time (min)	0.17	0.28	0.21	1.000

**Table 4:** Demonstration of median, interquartiles and p values of non-normally distributed continuous variables in the gene expression data in pre- and post-operative tissue and blood samples and control blood samples

	*PTEN* gene expression			Significance (Blood Samples) ‡	
	Blood (n=22)	Tissue (n=22)	Control (n=22)	PreOp vs. Control	PostOp vs. Control
**PreOp**	0.89 (0.47, 3.49)	0.83 (0.76, 1.50)	1.03 (0.41, 1.91)	0.814	0.742
**PostOp**	0.80 (0.20, 2.13)	1.55 (0.97, 2.23)			
**Sig. (p)** †	0.072	**0.053**	N.A.		

* Summarized using median (quartiles). N.A.: Not applicable.

† PreOp versus Post Op comparisons via Wilcoxon test.

‡ Versus control comparisons for blood samples via Mann-Whitney U test.

**Table 5:** Demonstration of median, interquartiles and p values of continuous variables that non-normally distributed in protein expression data in pre- and post-operative tissue samples

PTEN protein expression		
**n=8**	**P-PTEN**	**PTEN**
**PreOp**	0.66 (0.61, 1.14)	0.51 (0.47. 0.84)
**PostOp**	1.64 (1.23, 2.10)	1.67 (1.58. 2.28)
**Sig. (p)**	0.018	**0.018**

* Summarized using median (quartiles) and compared via Wilcoxon test.

## Discussion

Cardiovascular diseases (CVD), such as coronary artery disease (CAD), myocardial infarction (MI), and stroke are significant causes of mortality and morbidity in the general population worldwide.^2^CAD is the most common cause of death worldwide among cardiovascular diseases. PTEN plays a critical role in cardiomyocyte hypertrophy and survival. Therefore, the present study assessed the gene and protein expressions of PTEN in patients with CAD.

Mocanu et al. stated that the PI3K/AKT signaling pathway is vital in protecting the myocardium against ischemia-reperfusion injury.^16^They emphasized that the main factor that provides this activation is the PTEN gene. Additionally, PTEN may be essential in pathological conditions associated with ischemic heart diseases, such as diabetes and obesity.^12^Therefore, inactivation of PTEN may play an important role in pathological conditions associated with ischemic heart disease.^13^

Li et al. investigated PTEN expression to explore the relevance between the expression of PTEN and the development of CHD in myocardial tissue of 16 deceased patients through immunohistochemistry and qRT-PCR. PTEN protein expression in the myocardium was significantly lower in patients with coronary heart disease, as compared to the healthy control group. At the same time, there was no statistical difference in the expression of *PTEN* mRNA between the experimental and control groups. With these results, they concluded that PTEN might be involved in the occurrence and development of CHD.^19^Our study also showed that the *PTEN* gene and protein expression in myocardial tissue increased after bypass. We thought the results differed because postmortem tissue samples were used in the study mentioned above.

PTEN/PI3K signaling pathways play a crucial role in the pathogenesis of myocardial hypertrophy. In one study, it was found to be involved in myocardial remodeling in 39 patients with congestive heart failure (CHF). PTEN protein expressions in the congestive heart failure groups were lower than in the control group and were negatively correlated with cardiac function levels. Thus, they revealed that PTEN may well play a negative regulatory role in the myocardial remodeling process.^20^Cardiac hypertrophy, a general adaptive response of the heart, is a complex process in which many genes work in a coordinated manner. It can be stimulated in several ways, including the IGF-1/PI3K/Akt pathway. In the PI3K signaling pathway, PTEN can be a critical determinant of cardiomyocyte growth. Under certain conditions, it can cause apoptosis, prevent hypertrophy, and block growth factor signaling.^14^In our study, both PTEN genes and protein expressions in pre-operative myocardial tissues significantly increased after surgery. This result shows us repeatedly that PTEN plays an important role in repairing and enhancing cell survival, myocardial contractility, and cardiomyocyte viability in tissue damage. Considering all these effects, PTEN will provide an important prognostic marker in patients with CAD. One of the recent studies revealed that PTEN plays a role in the progression of myocardial infarction. It has been observed that serum PTEN levels are increased in patients with acute MI, suggesting that PTEN can be used as a predictive marker in MI.^21 , 22^One of the recent studies is Feng et al.’s study. In 2020, they found that the BPV as a PTEN inhibitor given to the mice improved the function of cardiac vessels after myocardial infarction. They thought that the PTEN inhibitor BPV could be a candidate therapeutic drug.^23^

The activity of PTEN can be reduced in two ways: one is achieved by enzymatic inactivation through phosphorylation or oxidation, and another by changing the balance between PTEN synthesis and degradation. The change in PTEN activity is important in maintaining balance in many cell types. It also plays a vital role in regulating the balance between survival and death in cardiomyocytes.^16^PTEN function is regulated by different post-translational modifications, such as phosphorylation, acetylation, ubiquitination, and oxidation. PTEN participates in the modulation and stability of its tumor suppressor functions. It also has six phosphorylation sites involved in subcellular compartmentalization.^8 , 24^The C-terminal region of PTEN is composed of 218 amino acids. It plays a role in regulating the stability and half-life of the molecule. This region is rich in phosphorylation sites, and phosphorylation of the PTEN C terminal has been reported to affect PTEN protein stability and function. Remarkably, the casein kinase 2 (CK2) phosphorylation sites in PTEN are conserved in species.^25^Our study also supports this idea. We showed that PTEN had increased phosphorylation from the point of S380 in the C tail. Phosphorylation of S380 residue inhibits PTEN’s catalytic activity and stabilizes the protein by blocking the productive association of the PTEN catalytic domain with membrane-localized PI(3,4,5)P3.^25^

We demonstrated a significant difference in the PTEN expression of myocardial tissues during the surgery. In our study, obtaining blood and tissue specimens at different surgery times was difficult, but it made our work different from others. Without the use of healthy controls, first, we compared the tissue and blood samples within the group by eliminating the individual differences and made it possible to show differences in expression levels on the same occasion. We then enrolled the control group to compare the expression levels of PTEN in the blood. The gene expression of *PTEN* in the blood may be reduced by epigenetics and other mechanisms. Epigenetic effects can alter the activity of PTEN. Some miRNAs can bind to the 3 ‘UTR of PTEN (such as miR-21), alter the proliferation of vascular endothelial cells via PI3K-Akt, and affect CAD.^26^Some previous studies^27^have suggested that PTEN is upregulated in peripheral blood mononuclear cells of CAD patients. This is consistent with the findings of Zhang et al.’s study on peripheral blood leukocytes,^28^and these study results support our study. Our results differed from the aforementioned study’s blood levels compared to the healthy controls. The same levels were detected in the control and the patients for PTEN in the blood.

TnI and TnT as protein markers of myocardial infarction were first proposed more than 20 years ago. Immunochemical measurement of TnI or TnT concentrations in the patient’s blood sidelined the traditional methods of diagnostics of myocardial infarction.^29^Troponin and CK-MB levels refer to necrosis and ischemia in cardiomyocytes. Cardiac-specific troponin T (cTnT) is elevated in the blood following cardiac injury.^30^The increase in clinical data is also related to the degree of ischemia. According to the literature, our study found that both CK-MB and troponin levels increased significantly after surgery. We also determined a correlation between CK-MB and TnT levels. Based on these findings, it would be helpful to determine the cause of the difference in troponin and CK-MB by planning more extensive and different studies to reveal the structural damage in myocardial tissue. Apart from the known functions of troponin, it has been reported to have a role in cell proliferation.^31^As mentioned above, the elevated levels of clinical biomarkers of the myocardium, both gene and protein expression of PTEN, were higher after the surgery. An increase in *PTEN* as a tumor suppressor due to the increase in TnT level may also be responsible for suppressing the proliferation of damaged cells.

As in many systems, miRNAs play an essential role in the cardiovascular system. In addition to controlling the functions of cells, such as cardiomyocytes, smooth muscle cells, fibroblasts, and endothelial cells, it is also crucial in the pathophysiology of diseases, such as myocardial infarction, hypertrophy, fibrosis, heart failure, arrhythmia, inflammation, and atherosclerosis. Therefore, investigating the miRNAs associated with *PTEN* will also benefit from expanding our study and acquiring new data. In this respect, our study is the first of its kind in the literature. However, when considering the unknowns regarding the PTEN pathway, planning new investigations in this area would be appropriate.

*PTEN* functions as a critical regulator of cardiomyocyte hypertrophy and survival. From this point of view, we investigated the *PTEN* gene and protein expressions in the blood and tissue samples. Studies using blood and myocardial tissues are rare in the literature, and it is challenging to conduct such studies. When we compared the *PTEN* gene expression in the blood, there was no difference between the PTEN expression before and after the surgery. No difference was found when the patients were compared with the control group. We observed increased protein and gene expression in tissue after surgery compared to before. The increase in PTEN expression in myocardial tissue after surgery compared to the preoperative sample repeatedly shows that PTEN plays a vital role in repairing tissue damage, healing, and increasing cell survival, myocardial contractility, and cardiomyocyte viability. Considering all these effects, the *PTEN* gene will enable us to take a critical prognostic measure in coronary artery disease in the following years. Molecular studies in this area are scarce, and we believe our findings about PTEN can lead to future studies. The limitation of this study is the small number of patients. In addition, our results can be supported by investigating the relationship between broader clinical parameters and PTEN levels.

## Conclusion

PTEN functions as a critical regulator of cardiomyocyte hypertrophy and survival. According to the results obtained from this study, it can suggested that the PTEN gene may be a marker in this disease group by expanding the number of patients for further investigations. However, when considering the unknowns regarding the PTEN pathway, planning new investigations in this area would be appropriate. Innovative studies may be planned with larger samples to conclude the real effect of PTEN activity on myocardium and accept it as a prognostic marker.
